# The impacts of visual Art Therapy for elderly with Neurocognitive disorder: a systematic review

**DOI:** 10.1590/1980-5764-DN-2021-0042

**Published:** 2022

**Authors:** Leonardo Brynne Ramos de Souza, Yasmin Cabral Gomes, Márcia Goretti Guimarães de Moraes

**Affiliations:** 1 Universidade Federal do Pará, Hospital Universitário João de Barros Barreto, Belém PA, Brazil.; 2 Universidade da Amazônia, Faculdade de Artes Visuais, Belém PA, Brazil.; 3 Universidade do Estado do Pará, Unidade de Ensino e Assistência em Fisioterapia e Terapia Ocupacional, Belém PA, Brazil.

**Keywords:** Art Therapy, Creativity, Neurocognitive Disorders, Terapia pela Arte, Criatividade, Transtornos Neurocognitivos

## Abstract

Aging is characterized by a reduction in physical, cognitive, and emotional functions, allowing multiple losses in the quality of life of the elderly. As a clinical situation that has a common association with pathological aging, neurocognitive disorder, previously named dementia, has become epidemiologically more relevant over the years. Thus, art therapy has recently emerged as an alternative technique for approaching these individuals, with the aim of improving cognitive, emotional, and quality of life aspects.

**Objective:**

The objective of this study was to analyze the effects of the application of art therapy based on visual creative processes on cognitive, emotional, and quality of life aspects in elderly people with neurocognitive disorder.

**Methods:**

This was a systematic review, carried out using CAPES, PubMed, SciELO, Bireme, PEDro, LILACS, and Scorpus databases between December 2020 and April 2021. Controlled and uncontrolled clinical trials were included in English, Spanish, French, and Portuguese, published between 1970 and 2021, with a focus on modalities of visual art production. The articles included at the end of the selection process were evaluated methodologically by the PEDro Scale of clinical trials.

**Results:**

A total of 14 articles were obtained at the end of the selection. Of note, 13 articles had a statistically significant outcome (p≤0.005) for quality of life, cognitive, and emotional functions after intervention with art therapy, being the most used interventions, such as drawing, paintings, and sculptures.

**Conclusions:**

The results show significant impacts after the application of art therapy in its visual modality. However, studies with greater methodological rigor are needed to strengthen the evidence presented.

## RESUMO.

O envelhecimento é caracterizado pela redução das funções físicas, cognitivas e emocionais, possibilitando múltiplas perdas na qualidade de vida do idoso. Como situação clínica que possui comum associação com o envelhecimento patológico, o transtorno neurocognitivo, anteriormente chamado de demência, vem-se tornando epidemiologicamente mais relevante com o passar dos anos. Desse modo, a terapia pela arte surgiu recentemente como técnica alternativa para a abordagem desses indivíduos, com o objetivo de melhorar o estado cognitivo e emocional e a qualidade de vida.

**Objetivo:**

Analisar os efeitos da aplicação da terapia pela arte baseada em processos criativos visuais nos aspectos cognitivos e emocionais e na qualidade de vida em idosos com transtorno neurocognitivo.

**Métodos:**

Trata-se de uma revisão sistemática, realizada nas bases de dados da Coordenação de Aperfeiçoamento de Pessoal de Nível Superior (CAPES), PubMed, Scientific Electronic Library Online (SciELO), Centro Latino-Americano e do Caribe de Informação em Ciências da Saúde (Bireme), Physiotherapy Evidence Database (PEDro), Literatura Latino-Americana e do Caribe em Ciências da Saúde (Lilacs) e Scorpus, realizada entre dezembro de 2020 e abril de 2021. Foram incluídos ensaios clínicos controlados e não controlados em inglês, espanhol, francês e português, publicados entre 1970 e 2021, com enfoque em modalidades de produção de arte visual. Os artigos incluídos ao fim do processo de seleção foram avaliados metodologicamente pela escala PEDro de ensaios clínicos.

**Resultados:**

Catorze artigos foram obtidos ao fim da seleção. Treze tiveram desfecho estatisticamente significativo (p=<0,005) para qualidade de vida, funções cognitivas e emocionais após a intervenção com arte-terapia, sendo as intervenções mais usadas: elaboração de desenhos, pinturas e esculturas.

**Conclusões:**

Os resultados apontam para impactos significativos após a aplicação da arte-terapia em sua modalidade visual. No entanto, são necessários estudos com maior rigor metodológico para fortalecer as evidências apresentadas.

## INTRODUCTION

During aging, a decline in physiological reserves, including those that make up the individual's cognitive performance, is expected. This process, called senescence, does not lead to functional losses. However, when old age is accompanied by impairments in the ability to perform activities of daily living (ADLs), then there is a picture of senility. In these cases, the neurocognitive disorder is included, previously named dementia^1^. The expected growth of elderly people diagnosed with this condition, as well as demographic aging, implies the adoption of multidisciplinary therapeutic interventions, which are necessary to approach these patients, considering the severity levels of the condition, for example^2^.

All historical documents confirm that the production of images, manual work, creation of stories, traditions, dances, and songs have always been a feature of life in society, as well as their use as escape instruments, entertainment, and even treatment^3^. Since the beginning of time, when men had not even perceived themselves as such, art was already existent and present. When we mention art as a whole, we bring and imagine works that portray a historical, classical, and critical perspective, but that was not always the case. Previously, art served as a way of recording what was around, the first glances and perceptions of the peoples to inhabit the earth, and the records that were found in caves, especially in the region of Europe around 16,000 BC, where primitive people shared their stories through drawings made with natural pigments, such as tree bark, fruits, and animal remains — called Prehistoric Art, also known as Rock Art^4,5^.

From this initial manifestation, humans began to feel the need for art in a constant and absolute way, either for their own records or to expose their ideas and thoughts, in addition to the continuous desire to share these reflections with their fellow men. Over time, art became indispensable and irreplaceable, present in all forms of poetic, reflective, and philosophical manifestations, as well as becoming synonymous with well-being, education, entertainment, and hobbies^6^. Because of this, a long social discussion was formed, considering artistic processes as methods to regenerate human health and well-being, especially in the past century. However, there are few literary productions about the real effectiveness of art therapy, as it would be called later^7^.

Art therapy is an umbrella term, which designates a group of human actions, of a mental integrative character, enhancing the individual, familiar, and communicative life of people through creative processes. It combines the principles of psychological theory and human experiences within a psychotherapeutic relationship^8^. As broad as this definition, the concept of health is defined by the World Health Organization (WHO) as a state of complete physical, mental, and social well-being, but not merely the absence of illnesses or diseases^9^. Bozcuk et al. cited the effects of art therapy on quality of life and the development of chemotherapy in patients diagnosed with cancer. In their results, art therapy was identified as a way to improve the quality of life of these patients, especially those who had no previous exposure to art therapy^10^.

In the literature review, Stuckey and Nobel selected the works containing approaches derived from the concept of art therapy and divided them into four main strands: music therapy, visual art, creative expression based on movement, and creative writing. Among the results, there was a clear indication that artistic engagement would trigger positive effects on the emotional health and cognition of study participants. However, the methodological quality of the published works would limit more solid conclusions^11^.

Art therapy involves sensory and intellectual stimulation, which leads to different impacts on the neuronal cell processes of healthy elderly people or those with early or mild cognitive impairments from Alzheimer's disease^12^. However, it is important to mention that neurocognitive disorder is a clinical syndrome marked by cognitive decline, leading to progressive impairment in the subject's functional capacity. Although the most common is the phenotype behind Alzheimer's disease, it can also be classified into vascular dementia, Lewy body dementia, frontotemporal dementia, and Parkinson's disease dementia^13,14^.

Physical exercise is an already consolidated therapeutic line for the prevention and promotion of health and independence in these cases. Physical therapy — through the application of exercises and other therapeutic lines — is widely indicated through clinical trials of strong methodological quality as a method of prevention against cognitive dysfunction^15^. However, the population with neurocognitive disorder is generally considered difficult to manage by physical therapists, who have prominent training or education in the area. Furthermore, the cognitive factor has been treated as a positive index for the gains with therapy, considering that the physical therapy gains in patients with neurocognitive dysfunction are significantly lower^16^.

Given the above, it is concluded that the elderly with neurocognitive disorder lose their functional capacity, in addition to presenting cognitive and emotional disorders and loss of their quality of life and that this is still a question of many studies that need to be answered. Thus, this systematic review aimed to analyze the effects of applying art therapy based on visual creative processes on cognitive, emotional, and quality of life aspects in elderly people with neurocognitive disorders.

## METHODS

This is a systematic review, including therapeutic intervention studies, carried out between February and August 2021, covering art therapy based on the execution of visual artistic creative processes to address neurocognitive disorders in elderly patients.

### Systematic Review Protocol

The research was carried out in the following databases: CAPES, PubMed, SciELO, Bireme, PEDro, LILACS, and Scorpus, between December 2020 and April 2021. Articles in English, Spanish, French, and Portuguese, published between 1970 and 2021, were included.

Controlled and uncontrolled clinical trials with elderly patients with neurocognitive disorder were included, excluding systematic review or literature articles, in vitro experimental studies, book chapters, and simple and expanded abstracts published in event proceedings. Studies with Alzheimer's disease, vascular dementia, advanced Parkinson's disease, Lewy body dementia, frontotemporal dementia, Huntington's disease, alcohol dementia (Korsakoff's syndrome), and Creutzfeldt-Jakob disease were included.

The included articles used some modality of visual artistic intervention, applied and developed by a professional with training or education in the area identified in this article. Interventions based on dance, music therapy, and other nonvisual procedures were excluded.

### Research strategies

The research was started in 6 different base data: CAPES, PUBMED, Scielo, Lilacs/Bireme and PEDro. It was only always used key-words created by the DeCs, but connecting all of them with boleans operators. The platform is being described in the Table 1.

**Table 1:** Descriptors used in the databases and the number of articles found in each database.

Database	Search research
CAPES	(terapia pela arte) E (demência)
	(criatividade) E (demência)
	(arteterapia) E (demência)
PUBMED	(art therapy) AND (dementia)
	(art therapy) AND (cognitive disorders)
	(art therapy) AND (cognitive disorders) OR (alzheimer)
	(art therapy) AND (delirium)
	(creative arts) AND (dementia)
	(creative arts) AND (cognitive disorders)
	(creative arts) and (cognitive disorders) OR (alzheimer)
	(visual arts) and (dementia)
	(art therapy) AND (alzheimer)
	(visual arts) AND (alzheimer)
	(creative arts) AND (alzheimer)
	(creativity) AND (dementia)
SciELO	(art therapy) AND (dementia)
	(art therapy) AND (dementia) OR (alzheimer)
	(art therapy) AND (cognitive disorders)
	(art therapy) AND (cognitive disorders) OR (alzheimer)
	(visual arts) AND (delirium)
	(terapia pela arte) e (demência)
	(criatividade) E (demência)
	(arteterapia) E (demência)
	(arteterapia) Y (demencia)
	(creatividad) Y (demencia)
	(arteterapia) Y (demencia)
LILACS/Bireme	(art therapy) AND (dementia)
	(art therapy) AND (dementia) OR (alzheimer)
	(art therapy) AND (cognitive disorders)
	(art therapy) AND (cognitive disorders) OR (alzheimer)
PEDro	(art therapy) AND (dementia)
	(art therapy) AND (alzheimer) OR (cognitive disorders)
	(art therapy) AND (cognitive disorders) OR (dementia)

### Selection process

Two independent researchers performed the search in different databases. To select the studies, the authors considered the title and abstract of the article, organizing the works by year of publication, language, and source database. A third researcher would be included if the first two found themselves in a moment of disagreement, deciding whether or not to exclude the material. Selected articles were grouped again, considering year of publication, language, source database, intervention, and outcome.

### Data analysis

**Figure 1:**
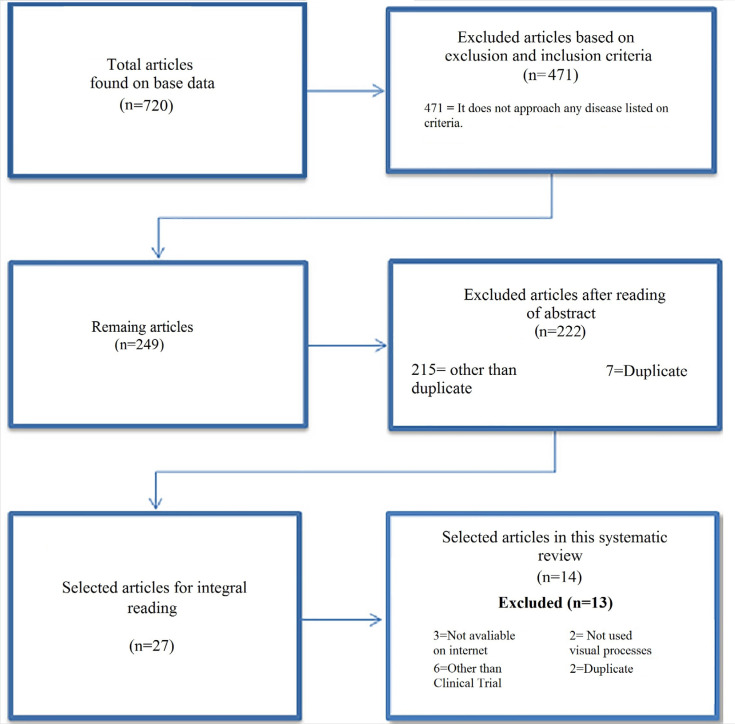
Flowchart of identified and selected studies based on database, criteria, and processes on research.

The process of analyzing the articles was divided into three phases: the first phase would be related to the inclusion and exclusion of articles based on the research inclusion and exclusion criteria; the second phase was the reading of titles and abstracts of each work, and the third and last phase was the full reading of the articles, as shown in Figure 1.

The articles were evaluated using the PEDro Scale, an instrument used to assess therapeutic intervention trials, with a high rate of reliability and scientific feasibility in therapeutic intervention studies. The articles that fit this scale had quality levels measured as follows: “low,” if they scored 0–3; “average,” if they scored 4–5; and “high,” if they scored 6–10.

## RESULTS

### Data analysis

**Figure 2:**
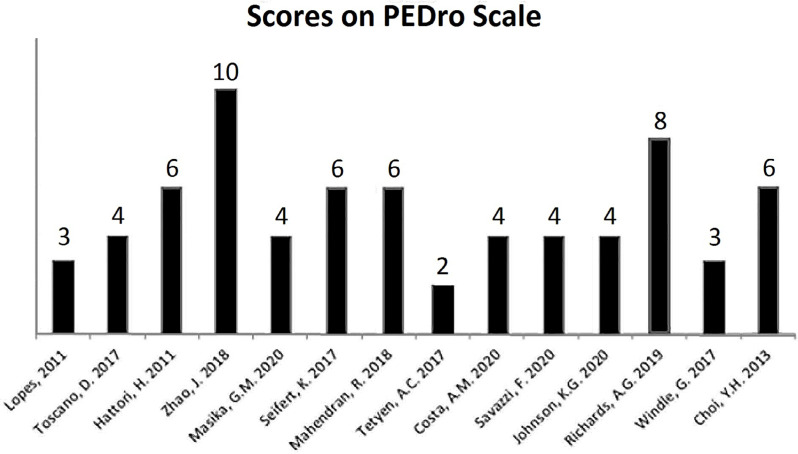
Percentage obtained using the PEDro Scale for the selected articles.

A total of 14 articles were selected for the systematic review, whose authorship data, year of publication, study method, evaluation instruments, and results are shown in Table 2. The articles were evaluated by using the PEDro Scale, and their scores are shown in Figure 2.

**Table 2:** Bibliographic sources identified, place of study, type of study, sample, evaluation protocol, intervention, and outcomes.

Authors	Year	Study type	Art therapy strategy	Assessment instruments	Results
Lopes^17^	2011	Noncontrolled clinical trial	Drawing, painting, and image cutting	Rey's Complex Figure Test Mini-Mental State Examination (MMSE)	n=6 Rey's Complex Figure Test: 16.8% increase in overall post-intervention score MMSE: 30 and 33.3% increase in the score in the Attention and Calculation items
Toscano et al.^18^	2017	Controlled clinical trial	Drawing, painting, and image cutting	Beck Depression Inventory-II (BDI-II) MMSE Dartmouth COOP/WONCA Functional Health Rating Scale (El COOP/WONCA)	n=32 Control group (CG): n=16 Intervention group (IG): n=16 Significant improvement in the Student's *t*-test in: BDI-II: −3.47 MMSE: −3.25 Feelings (t=-3.22) and Social Activities (-5.05)
Hattori et al.^19^	2011	Controlled clinical trial	CG: drawing, painting, and image cutting GI: math calculation involving multiplication and addition	MMSE Wechsler Memory Scale – Revised (WMS-R) Geriatric Depression Scale (GDR) Apathy Scale (Japanese version) SF-8	n=39 CG: n=19 IG: n=20 GI: MMSE: (significant difference compared to the CG, p=0.0015) Apathy Scale: (significant improvement compared to the CG, p=0.0014) MCS-8: (p=0.38, OR=5.54)
Zhao et al.^20^	2018	Controlled clinical trial	IG: warm-up by interaction game, drawing, and painting CG: occupational rehabilitation (exercises by lens, cognitive, and speech training strategy)	Changsha-Chinese version of the Montreal Cognitive Assessment (MoCA) Chinese version of the Auditory Verbal Learning Test (CVAVLT) Chinese version of the Category Verbal Fluency Test (CVCVFT) Digital Span Test (DST) Chinese version of Trail Making Test A (TMT-A) and Trail Making Test B (TMT-B) Chinese version of the Activities of Daily Living (CVADL) scale	n=93 CG: n=45 IG: n=48 Both groups showed good post-intervention results IG: greater benefits than CG on all instruments MOCA: F=21.24, p<0.001 CVAVLT memory domain – memory: immediate: F=4.81, p=0.023 and daily F=3.98, p=0.012 CVCVFT language function domain: F=3.91, p=0.017 Cognitive function: F=23; 35. p<0.001 Attention domain: F=3.29, p=0.030 Memory satisfaction (MSQ: F=18.39, p<00001)
Masika et al.^21^	2020	Controlled clinical trial	IG: drawing, painting, and artistic expression. CG: educational guidelines in health	Montreal Cognitive Assessment – 5-minute protocol (MOCA-5min) Geriatric Depression Scale – Short Form (GDS-SF)	n=39 IG: n=21 CG: n=18 MOCA: IG and CG had significant improvements in cognitive scores over time GDS-SF: IG shows more intense improvement in scores than CG (β=-3.0, SE=0.822, p<0.001)
Seifert et al.^22^	2017	Controlled clinical trial	IG: sculpture assembly activities CG: painting, singing, and playing	MMSE Neuro-Psychiatric Inventory (NPI)	n=12 CG: n=6 IG: n=6 MMSE: There were no significant differences between CG and IG NPI: There were no significant differences between CG and IG
Mahendran et al.^23^	2018	Randomized controlled trial	IG I: (art therapy) IG II (Musical reminiscence activities) CG: did not receive therapeutic intervention	Rey's Verbal Auditory Learning Test (RAVLT) Third edition of the Wechsler Adult Intelligence Scale Visuospatial Skill Tests Procedural and Attention Tests Color Test GDS Geriatric Anxiety Inventory Analog visual scale Telomere Size Analysis	n=68 IG I: n=22 IG II: n=24 CG: n=22 Wechsler Scale: Higher mean scores in the CG for the items List Learning (CI=90%, (d)=0.542) and Digit Span Forward (d=0.0991; CI=90%; p=0.028) IG (art therapy): good results in the items Delayed Recall, Recognition Trials, Black Design, and Coolor Trials 2, but not statistically significant and higher than the CG. GDS: IG (art therapy) and IG II (musical reminiscence activities) had a decrease in the scale, but not statistically significant (p≥0.005) Telomere size: increase in telomere size (p=0.03) in the IG (art therapy) and IG II (musical reminiscence activities, p=0.076)
Tietyen and Richards^24^	2017	No controlled clinical trial	Decoration, collage, painting, ceramics, photography, printing, and photo mounting	Alzheimer's Disease Quality of Life Scale (QoL-AD) Rosenberg High Estimate Scale Smiley Face Mood Assessment Systematic Observation	n=8 QoL-AD: Improved index of all scale scores Rosenberg High Estimate Scale – Improvement in the index of all scale scores Smiley Face Mood Assessment – Improved index of all scale scores
Costa et al.^25^	2020	Clinical controlled trial	Collage, painting on canvas, watercolors, dry pastel, and drawing. IG: received guidance from researchers CG: did not receive guidance from researchers	MOCA Verbal Phonemic Fluency Clock-Drawing Test TMT-A and TMT-B Stroop Test Wechsler Memory Scale III Hospital Depression and Anxiety Scale (HADS) World Health Organization Quality of Life Questionnaire (WHOQOL-Bref)	n=12 CG: n=3 IG: n=9 MOCA: negative medians in both groups TMT-A and TMT-B: negative medians in both groups Stroop Test: negative medians in the Unguided Group WHOQOL-Bref: negative medians in the Guided Group
Savazzi et al.^26^	2020	Randomized controlled trial	IG: Painting, drawings, and creative stimulation through audiovisual means CG: Did not receive treatment	QoL-AD Neuropsychiatric Inventory (NPI) The Cognitive Subscale of the Alzheimer's Disease Rating Scale (ADAS-cog)	n=20 CG: n=10 IG: n=10 QoL-AD: improvement in IG's quality of life NPI: improvement in IG scores (frequency, p=0.014; nuisance, p=0.006). ADAS-cog: statistically significant improvement in quality of life in IG compared to CG, p<0.001)
Johnson et al.^27^	2020	Randomized controlled trial	IG: drawing and collage GC: waiting list	MOCA Backward Digit Span Test	n=53 CG: n=26 IG: n=27 MOCA (General Cognition): IG had no significant differences from the CG (GI: M=0.37; SD=2.7) MOCA (Delayed Recall): authors claim that it is impossible to declare whether or not there were statistically significant differences IG: M=-0.093; SD=0.68; CG: M=0.15; SD=0.61 Backward Digit Span Test: there were no significant differences between IG: M=-0.037; SD=1.3 and CG: (M=0.040; SD=1.8)
Richards and Tietyen^28^	2019	Randomized controlled trial	Decoration, collage, painting, ceramics, photography, printing marks, and photo mounting	Rosenberg's Self-Esteem Scale QoL-AD Functional Assessment Questionnaire	n=27 CG: n= 12 IG: n=15 Rosenberg High Estimate Scale: significant post-intervention differences in IG compared to CG (p=0.028) QoL-AD: better results were found in the scores, but statistically nonsignificant results after the intervention compared to CG (p=0.082); FAQ: statistically nonsignificant post-intervention results (p=0.17);
Choi and Jeon^29^	2013	Quasi-experimental study	Drawing, painting, and decorating	MMSE-Korean version (MMSE-KC) GDS-SF Quality of Life Questionnaire derived from the Ministry of Health of South Korea	n=64 CG: n=30 IG: n=34 MMSE-KC: IG had statistically significant improvements compared with CG (t=6.72, p<0.001) GDS-SF: IG had statistically significant improvements compared with CG (z=-4.55, p<0.001) Quality of Life: IG patients improved more significantly than CG patients (t=-2.39, p<0.05)
Windle et al.^30^	2017	Longitudinal study	Elaboration of sculptures, paintings, and drawings	The Greater Cincinnati Chapter Well Being Observation Tool (GCCWBOT) Quality of Life and Dementia Scale (DEMQOL) Holden Communication Scale	n=125 GCCWBOT: Significant improvements were found in the “negative affections” (p=0.001) and “interest” (p≤0.001) domains. No changes to normality and disengagement items. DEMQOL: no significant changes were found Holden Communication Scale: results indicate worsening of social behavior and communication with the person with neurocognitive disorder

According to the PEDro Scale score, three articles were classified into low-quality essays^1,3,13^, five articles had a reasonable methodological quality index^2,5,9–11^, and six articles had high-quality numbers methodological quality index^4,6–8,12,14^. There was a mean score of 5.78, with a standard deviation of ±3.30, considering a maximum score of 10 among the works included. Among the main difficulties of the studies, using the PEDro Scale assessment items, the following were registered: the absence of concealment of the allocation and randomization process of patients; presence of blinding of patients, therapists, and study evaluators; and specification of eligibility criteria for admission to the respective studies.

In cognitive analysis instruments, five studies applied the Montreal Cognitive Assessment (MOCA) Scale^4,5,6,8,10^. Another five studies applied the Mini-Mental State Examination (MMSE)^1–3,7,13^. To assess the quality of life and emotional aspects, the Geriatric Depression Scale (GDS) was the most used questionnaire^3,5,7,13^, followed by the Neuropsychiatric Inventory (NPI)^6,9^.

The most used interventions were drawing^1–14^, painting^1–3,5–14^, image cutting^1–3^, and constructing sculptures or ceramics^6,8,12^.

### Outcomes

The articles by Seifert^22^ and Johnson^27^ were the only ones that did not find benefits in the application of art therapy based on visual creative processes, whether it is in cognitive aspects (MMSE), in the evolution of the severity of dementia symptoms (NPI)^22^ or in the quality of life (World Health Organization Quality of Life Questionnaire or WHOQOL), and in the execution of tasks requested during the application of the study (Clock-Drawing test, Sloop test and MOCA). In Johnson's article, the group that received the intervention did not produced any different results from the control group, which was composed of patients on the waiting list.

Mahendran^23^ did not obtain statistically significant results between the pre- and post-intervention periods in the Wechsler Scale, although it computed positive oscillations in the data from the instruments used and from the GDS. The data are in agreement with the article by Hattori,19 which also used the Wechsler Scale as a screening tool and collected statistically significant results, this time, between the intervention group and the control group.

Some articles that applied the MOCA^20,21,25,27^ identified positive variations in the data collected before and after the intervention with art therapy (p≤0.005), whereas others applied the MMSE efficient in improving the quality of life, cognitive functions, and emotional condition of the subjects who were admitted to it. The Alzheimer's Disease Quality of Life Scale (QoL-AL) was applied in three articles^24,26,28^, capturing statistically significant improvements when comparing the intervention groups with the control groups. Most of the results show that the art therapy strategies were efficient in improving the quality of life, cognitive functions, and emotional condition of the subjects who were admitted to it.

Some articles that applied the MOCA^4,5,8,11^ identified positive variations in the data collected before and after the intervention with art therapy (p≤0.005), whereas others applied the MMSE efficient in improving the quality of life, cognitive functions, and emotional condition of the subjects who were admitted to it. The Alzheimer's Disease Quality of Life Scale (QoL-AL) was applied in three articles^9,10,12^, capturing statistically significant improvements when comparing the intervention groups with the control groups. Most of the results show that the art therapy strategies were efficient in improving the quality of life, cognitive functions, and emotional condition of the subjects who were admitted to it.

## DISCUSSION

According to the vast majority of studies collected for this research, the inclusion of art therapy in health treatments brought numerous benefits, such as improvements in motor coordination, perception, well-being, memory and depressive symptoms. The most used activities are drawing and painting^34^. However, artistic production can be made possible through materials and techniques that involve the use of colored pencils, crayons, gouache paints and, in some cases, the use of modeling clay, plaster and sculptures, such as was found in the articles of this review^35^.

The article by Lopes et al. suggests that the art-therapeutic strategies brought improvement in the MMSE indexes and in the King's Figure Test in its intervention group. However, the results of this work also make it possible to understand that this intervention adds up in another way to issues of the individual's experience, emphasizing the chance that the elderly have to express their particularities through expression and creativity. Toscano et al., along the same lines, emphasizes in his study that the assessment of these variables should not disregard the patient's personality characteristics, such as the presence of anxiety, emotions, fears and insecurities. Thus, the multidisciplinary integration behind the application of this intervention is of paramount importance, in order to expand the interpretation of the data, through discussions between physicians, psychologists, occupational therapists, physiotherapists, art professionals, etc. Allied to this, the presence of the word “expression” is remarkable, since it is through its means that human beings come to better understand and understand the other. The conclusion obtained by Toscano takes into account the appearance of depression, loneliness and sadness more frequently in the elderly, who end up suffering from anxiety, stress and nervousness when diagnosed with Neurocognitive Disorder. These emotional stress events complicate the patient's recovery process, prolonging their suffering and accentuating their neuropsychiatric disorders.36

The studies by Zhao and Hattori et al. mention that the strategies based on visual creative processes brought superior results in their evaluation instruments in relation to traditional cognitive rehabilitation, advocating the incorporation of activities based on narration and imagination in traditional interventions. Hattori also affirms that there was no disparity between the results after applying an intervention with painting or drawing, and that the strategy that receives the most satisfaction from patients should be preferred.

Masika et. Al found better answers regarding the quality of life of the elderly in the Geriatric Depression Scale. Patients in this study reported improvement in the feeling of empowerment and memory, psychosocial and cognitive health, sleep pattern and depressive mood, although they did not have better results compared to the control group in the MOCA. By the way, these are typical manifestations of the neurocognitive picture^35^. The target audience of this article was elderly people with a low level of education, including illiterate people, who had their first contact with writing, decoding and production of symbols or images with this study. Thus, the psycho-emotional response found in this audience is in line with what has been previously described in other works in the literature.

The study by Mahendran et al., on the other hand, points out that the artetherapeutic strategy was able to reverse the cellular scenario of inflammation around the telomeres, which is associated with reduced cognitive performance and systemic aging in general. However, there were no statistically significant differences between the groups that received the intervention, indicating the need for new studies with larger populations to be carried out to consolidate new results. It is noteworthy that no adverse effects were found in this and other studies associated with the intervention.

Tietyen et al., on the other hand, looked to the visual arts for new ways of learning to improve cognition, coordination, and emotional state, with the aim of re-socializing them. Interventions based on decoration, collage, painting, ceramics, photography, impression marks and assembly improved communication, fine motor skills, coordination and oculomotor skills, giving them independence in Activities of Daily Living (ADL). Likewise, Savazzi applied activities such as drawing, painting and creative stimulation through the audiovisual system in elderly people diagnosed with Alzheimer's Disease. Using the QoL-AD, NPI and ADAS-cog in this group, the group that received intervention in the disease achieved significant improvements in language, communication and cognitive function. The authors also mention the high adherence that the treatment had by the patients, again emphasizing the satisfaction and trust that people had in the treatment that was proposed to them.

Going against what was mentioned in other studies, Johnson et al. did not observe any statistically significant effect associated with the artetherapeutic intervention in an intervention group, comparing it with the control group, populated by patients on the waiting list. The instruments used to measure the variables were: MOCA and the Backward Digit Span, the same ones used in other works covered by this review. The authors say it is extremely unlikely that quantifiable cognitive benefits will occur with art training in a population with Neurocognitive Disorder, stating that they are confident in the hypothesis that the measures selected by the group are not the cause of the null results. However, researchers still elucidate the importance of promoting the artistic potential of these patients.

Thus, it is concluded that further efforts with greater methodological rigor are still needed to consolidate, in fact, the cognitive effects of the art-therapeutic approach in the post-intervention, given the greater instability of studies in the collection of this item. Furthermore, art therapy based on visual creative processes is shown in the observed data as a reliable proposal for improving the quality of life and self-knowledge of those who apply it.
